# Evaluating risk factors of embolism in patients with cardiac myxoma: A systematic review and meta-analysis

**DOI:** 10.1016/j.ahjo.2025.100559

**Published:** 2025-05-29

**Authors:** Muhammad Ahmad Qureshi, Danyal Bakht, Omair Ahmed, Shahan Haseeb, Kartik Gupta, Omar Baqal, Maaz Amir, Khawar Ali, Mirza Muhammad Hadeed Khawar, Muqaddas Hussain, Luqman Munir, Hussein Othman

**Affiliations:** a Henry Ford Jackson Hospital, Jackson, MI, USA; b King Edward Medical University, Lahore, Punjab, Pakistan; c Northwell Health System, NY, New York, USA; d Henry Ford Hospital, Detroit, MI, USA; e Mayo Clinic, Phoenix, AZ, USA; f Services Institute of Medical Sciences, Lahore, Punjab, Pakistan

**Keywords:** Embolism, Risk factors, Cardiac Myxoma

## Abstract

**Background:**

Cardiac myxomas (CM), the most common primary cardiac tumors, can cause embolism in about 40 % of cases, making it crucial to identify risk factors for guiding clinical decisions.

**Objectives:**

In this meta-analysis, we studied the risk factors associated with embolism among patients with cardiac myxomas.

**Methods:**

A comprehensive search was conducted across PubMed, Embase, and Cochrane Library from their inception until May 2023. Statistical analyses were performed using Cochrane's RevMan 5.4 software. For each risk factor, the pooled odds ratio or mean difference was calculated along with the corresponding 95 % confidence interval.

**Results:**

This meta-analysis incorporated 18 studies with 2601 patients, of whom 525 (20.1 %) experienced embolism. Significant risk factors included hypertension (*p* = 0.001), NYHA I/II (*p* = 0.03), irregular tumor surface (*p* < 0.01), hyperlipidemia (p < 0.01), coronary artery disease (*p* = 0.01), elevated mean platelet volume (*p* = 0.02), and high tumor mobility (p < 0.01), while female gender (p = 0.03) was linked to reduced risk. Smoking, atrial fibrillation, tumor size, age, BMI, diabetes, LVEF, and LAD were not significantly associated with embolism (*p* > 0.05).

**Conclusion:**

This analysis is the first to highlight significant pooled outcomes for gender, hyperlipidemia, coronary artery disease, mean platelet volume, and tumor mobility. Patients with these risk factors may benefit from early evaluation and surgery to reduce embolism risk. Statistical analyses were performed using RevMan 5.4, with pooled odds ratios or mean differences calculated alongside 95 % confidence intervals.

## 1 Introduction

Cardiac myxomas (CM) make up almost 50–70 % of the primary cardiac tumors [[1], [2], [3], [4]]. They are generally benign in nature, but they can lead to significant clinical complications. These complications include obstruction of cardiac blood flow, preoperative embolization, and various constitutional symptoms [5]. Embolization, a major and potentially fatal complication, occurs in about 40 % of patients with CM [6]. Cerebral embolisms, which account for majority of embolic events in these patients, often presents as acute stroke symptoms [7]. In addition to cerebral embolism, peripheral embolism may affect the extremities, visceral organs, and coronary arteries, while right sided myxomas are particularly associated with pulmonary embolism [7,8].

Understanding the risk factors for embolism in CM is critical for guiding clinical decision-making, particularly in timing surgical intervention and managing postoperative care. Several studies have attempted to identify the risk factors for embolism in patients with CM. A meta-analysis published previously aimed to pool these studies to draw more definitive conclusions [9]. However, the exact risk factors remain controversial, and the previous meta-analysis missed some relevant studies, particularly newer research published since its release. In light of these gaps, our objective is to conduct an updated meta-analysis to incorporate the latest evidence and determine whether new data alters the understanding of risk factors for embolism in CM patients. This updated review aims to provide a more comprehensive and current assessment of the issue.

## 2 Materials and methods

### 2.1 Search strategy

The study was conducted in accordance with the PRISMA (Preferred Reporting Items for Systematic Reviews and Meta-Analyses) guidelines [10]. A systematic literature search was conducted May 2023 on the following databases: PubMed/MEDLINE, Cochrane, Embase and ScienceDirect. The search strategy is reported in the supplementary file, and contains keywords like ‘myxoma’, ‘risk factor’ and ‘embolism’. Additionally, further studies were identified through a manual review of the reference lists of relevant meta-analyses on the topic [9] and by going through the bibliography of the studies identified through initial literature search. Notably, four studies conducted in China that were included in the previous meta-analysis could not be located in any online databases, with two of them being dissertations. Efforts to obtain the full manuscripts by contacting the authors were unsuccessful. Detailed references of those studies are provided in the supplementary file.

### 2.2 Eligibility criteria

The included study population comprised of adult patients diagnosed with CM on post-surgical histopathological evaluation. The included original research studies divide CM patients into embolism and non-embolism groups to evaluate and compare at least one risk factor. Excluded studies included abstracts, letters to editor, reviews, or studies that lacked division into embolic and non-embolic groups or had incomplete data.

### 2.3 Data extraction and quality assessment

Two authors screened the titles and abstracts of all retrieved records to identify studies that potentially met the eligibility criteria. Full texts for these studies were independently assessed by both reviewers for inclusion based on predefined eligibility criteria. Discrepancies were resolved through consensus and deliberation by a third reviewer. The extracted data included study identifiers, study characteristics, demographic data, patient and tumor characteristics, and relevant clinical patient data. The NYHA class was dichotomized into two groups: I/II versus III/IV. Tumor surface characteristics were classified as irregular or regular. Irregular surface characteristics encompassed features such as gelatinous consistency, papillary morphology, polypoid appearance, and other atypical types. Detailed study-specific data for this classification are provided in the supplementary file. Tumor size was determined by its greatest dimension in centimeters. Tumor mobility was categorized as either high or low, indicating degrees of movement within the heart. The location of the tumor was differentiated between those in the left atrium versus other areas. Additionally, tumor appearance was classified based on whether it was pedunculated or sessile.

The quality of the studies was assessed using the Newcastle-Ottawa Scale (NOS) [11], which was independently applied by two authors. The NOS evaluates critical components of cohort studies, specifically focusing on selection, comparability, and outcome assessment. Details are provided in the supplementary file.

### 2.4 Statistical analysis

Statistical analysis of the data was conducted using Review Manager 5.4. The odds ratio (OR) or mean difference (MD) along with their respective 95 % confidence intervals (CI) were calculated. The choice between fixed-effect (FE) and random-effect (RE) models was determined based on the heterogeneity among studies, assessed using Cochran's Q test and chi-square (I^2^) statistic. Heterogeneity levels were categorized as low (I^2^ ≤50 %) or high (I^2^ ≥ 50 %). A RE model was employed when substantial heterogeneity (I^2^ > 50 % or *P*-value <0.05) was identified; otherwise, a FE model was utilized. A sensitivity analysis was performed when significant heterogeneity (I^2^ > 75 %) was observed. Publication bias was evaluated using Egger's test. All statistical analyses were two-tailed, with significance determined at a threshold of *P* < 0.05.

## 3 Results

### 3.1 Selected studies and study characteristics

**Fig. 1:**
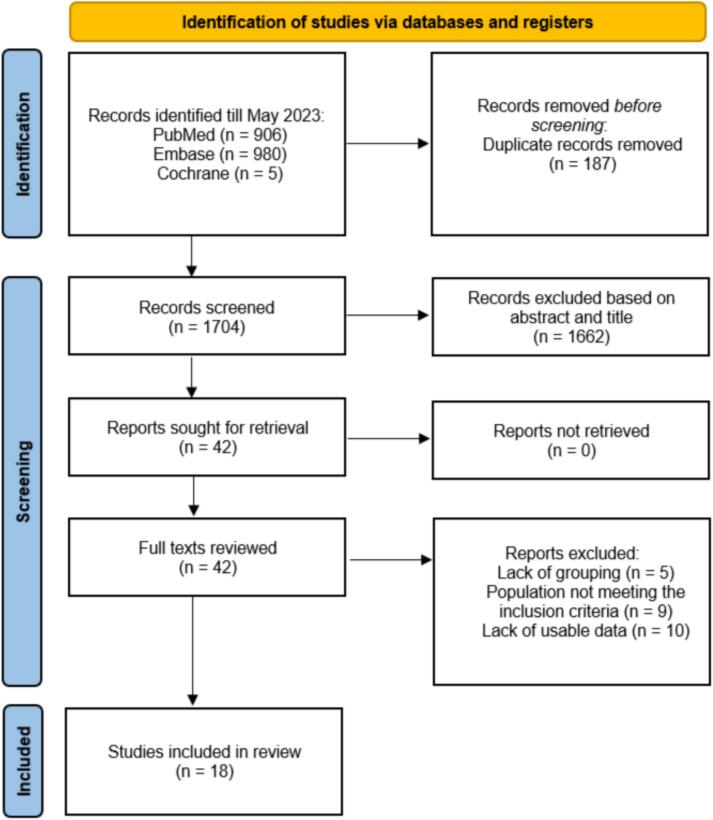
PRISMA flow diagram.

18 retrospective cohort studies, encompassing a total of 2601 participants were included. The detailed study selection process is illustrated in Fig. 1, and the key characteristics of the studies are summarized in Table 1 (included in supplementary file 1). Participants were categorized into embolic (*n* = 525, 20.18 %) and non-embolic (*n* = 2076, 79.81 %) groups across all included studies. Studies were conducted in seven countries: China, Turkey, Korea, Spain, Japan, USA, and Argentina.

### 3.2 Risk of bias in individual studies

Utilizing the NOS, the evaluation revealed that most studies were of high quality, with 9 out of 18 receiving 8 stars. The remaining 9 studies were rated at 7 stars, signifying strong quality with some minor limitations. The detailed scores for individual studies selected for risk of bias assessment have been reported in supplementary file.

### 3.3 Statistical analysis of risk factors

In this meta-analysis, 29 risk factors for embolism in patients with CM were assessed using both Fixed Effects and Random Effects models. These risk factors were grouped into baseline characteristics, serum and blood profile, and tumor characteristics. Table 2 presents the effect size, *P*-value, and heterogeneity for each risk factor. The funnel plots, illustrating the publication bias for each of the statistically significant risk factor have been shown in the supplementary file.

**Table 2:** Risk factors evaluated in the meta-analysis effect sizes, *P* values and heterogeneity across risk factors (FE: Fixed Effect; RE: Random Effect; OR: Odds Ratio; MD: Mean Difference; Ph: P value of heterogeneity). P-values <0.05 are highlighted by bold characters.

Risk factor	No. of studies	Effect model	MD/OR	I^2^ (%)	Ph	Effect size (95 % CI)	P value
Smoking	8	FE	OR	0	0.44	1.05 [0.79, 1.39]	0.74
Mobility	2	FE	OR	47	0.17	3.83 [2.45, 5.99]	**<0.00001**
Prolapsing nature	2	FE	OR	0	0.72	5.10 [0.97, 26.71]	0.05
Tumor size	10	RE	MD	86	<0.01	−0.07 [−0.76, 0.62]	0.85
Pedunculated appearance	5	FE	OR	0	0.89	1.18 [0.80, 1.75]	0.41
MPV	3	FE	MD	0	0.87	0.35 [0.06, 0.64]	**0.02**
NYHA I/II	04	RE	OR	55	0.08	2.20 [1.07, 4.50]	**0.03**
Diabetes	09	FE	OR	3	0.41	1.11 [0.80, 1.56]	0.53
Atrial fibrillation	09	FE	OR	28	0.20	1.27 [0.90, 1.81]	0.17
VHD	05	FE	OR	0	0.89	0.72 [0.45, 1.15]	0.17
Tumor hemorrhage	03	FE	OR	11	0.33	1.69 [0.74, 3.86]	0.21
Hypertension	09	FE	OR	0	0.50	1.52 [1.18, 1.96]	**0.001**
Hyperlipidemia	08	FE	OR	17	0.30	2.39 [1.59, 3.60]	**<0.0001**
Alcohol consumption	03	FE	OR	0	0.73	1.53 [0.74, 3.15]	0.25
CAD	06	RE	OR	73	<0.01	3.25 [1.31, 8.08]	**0.01**
LDL	03	RE	MD	87	<0.01	−0.98 [−30.41, 28.46]	0.95
Age	16	RE	MD	81	<0.01	−2.12 [−5.16, 0.91]	0.17
Attachment size	2	RE	MD	80	0.03	0.02 [−0.24, 0.29]	0.87
BMI	3	RE	MD	94	<0.01	0.78 [−1.77, 3.33]	0.55
Female gender	15	FE	OR	24	0.18	0.77 [0.62, 0.95]	**0.02**
Hb	3	RE	MD	76	0.02	0.32 [−0.84, 1.47]	0.59
Irregular surface	14	RE	OR	50	0.02	3.61 [2.35, 5.56]	**<0.01**
LAD	5	RE	MD	96	<0.01	2.42 [−2.31, 7.14]	0.32
LVEF	8	FE	MD	0	0.43	0.57 [−0.12, 1.26]	0.10
Platelet count	6	RE	MD	90	<0.01	7.07 [−28.69, 42.83]	0.70
Renal disease	3	FE	OR	0	0.81	0.59 [0.13, 2.77]	0.51
WBC count	5	RE	MD	51	0.08	−0.22 [−0.97, 0.53]	0.56
Left atrial location	8	FE	OR	0	0.49	1.21 [0.84, 1.74]	0.30

#### 3.3.1 Baseline characteristics

**Fig. 2:**
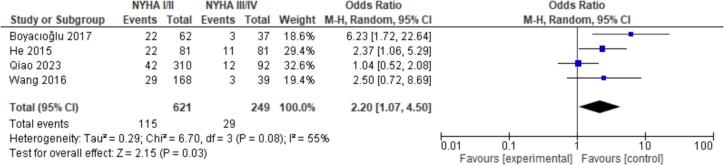
Forest plot for NYHA I/II between embolism vs nonembolism groups.

**Fig. 3:**
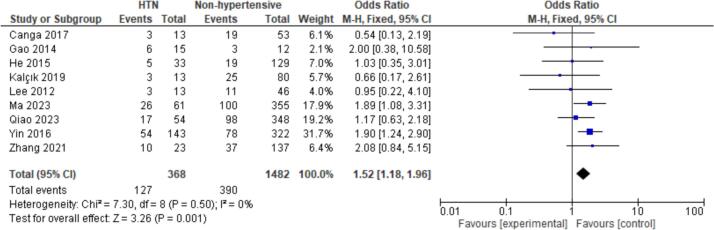
Forest plot for hypertension between embolism vs nonembolism groups.

**Fig. 4:**
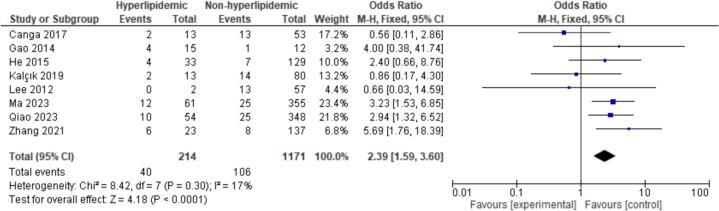
Forest plot for hyperlipidemia between embolism vs nonembolism groups.

**Fig. 5:**
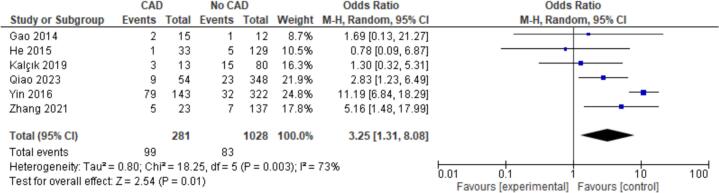
Forest plot for CAD between embolism vs nonembolism groups.

**Fig. 6:**
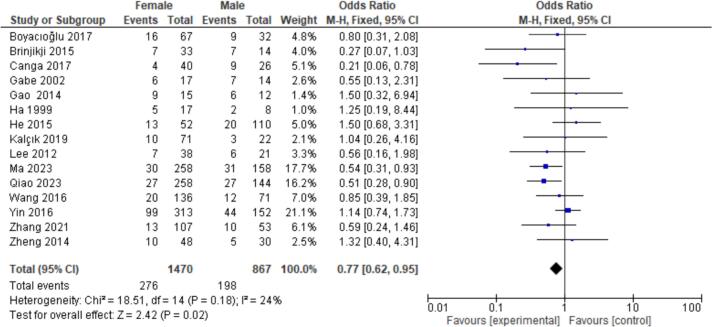
Forest plot for female gender between embolism vs nonembolism groups.

15 patient characteristics were evaluated. Statistically significant associations were found with NYHA I/II (OR = 2.20, CI = 1.07–4.50, P = 0.03), Hypertension (OR = 1.52, CI = 1.18–1.96, P < 0.01), Hyperlipidemia (OR = 2.39, CI = 1.59–3.60, *P* < 0.01), Coronary Artery Disease (CAD) (OR = 3.25, CI = 1.31–8.08, P = 0.01), and Female Gender (OR = 0.77, CI = 0.62–0.95, P = 0.02). Non-significant findings were observed for Smoking, Diabetes, Atrial Fibrillation, Valvular Heart Disease (VHD), Alcohol Consumption, Age, Body Mass Index (BMI), Left Atrial Diameter (LAD), Left Ventricular Ejection Fraction (LVEF), and renal disease (Fig. 2, Fig. 3, Fig. 4, Fig. 5, Fig. 6).

#### 3.3.2 Tumor characteristics

**Fig. 7:**
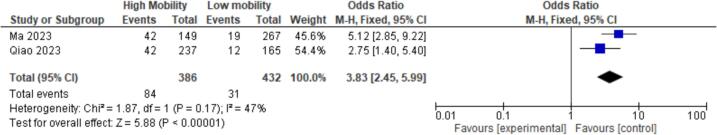
Forest plot for tumor mobility between embolism vs nonembolism groups.

**Fig. 8:**
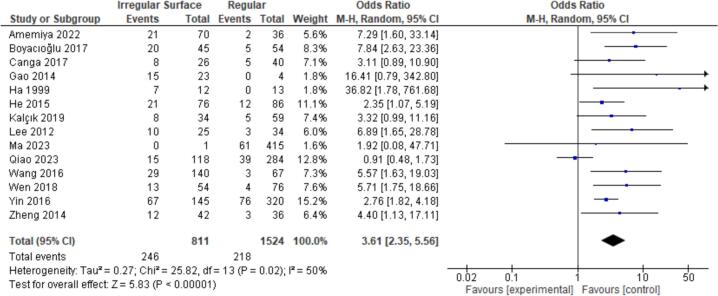
Forest plot for irregular tumor surface between embolism vs nonembolism groups.

8 tumor characteristics were evaluated. Statistically significant associations were found with High Tumor Mobility (OR = 3.83, CI = 2.45–5.99, *P* < 0.00001) and Irregular Surface (OR = 3.61, CI = 2.35–5.56, P < 0.00001). Non-significant findings were observed for Prolapsing Nature, Tumor Size, Pedunculated Appearance, Tumor Hemorrhage, Left Atrial Location, and Attachment Size (Fig. 7, Fig. 8).

#### 3.3.3 Hematological characteristics

**Fig. 9:**
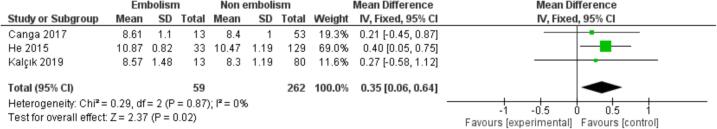
Forest plot for MPV between embolism vs nonembolism groups.

Five hematological characteristics were assessed. Among these, a high Mean Platelet Volume (MPV) was identified as statistically significantly associated with embolism (MD = 0.35, CI = 0.06–0.64, *P* = 0.02) (Fig. 9). However, no significant associations were found for Low-Density Lipoproteins (LDL) or White Blood Cell (WBC) Count.

### 3.4 Sensitivity analysis

**Fig. 10:**
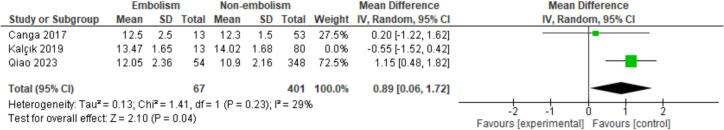
Forest plot for Hb between embolism vs nonembolism groups, leave-one-out analysis performed excluding Kalcik 2019 [13].

**Fig. 11:**
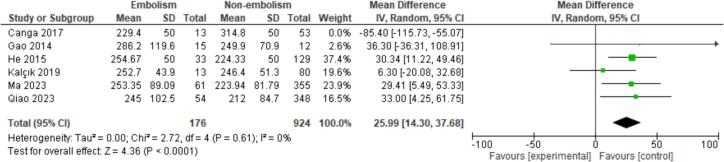
Forest plot for mean platelet count between embolism vs nonembolism groups, leave-one-out analysis performed excluding Canga 2017 [14].

A sensitivity analysis identified substantial heterogeneity across several variables, with risk factors showing >75 % heterogeneity according to Higgins & Thompson's I^2^ statistic [12]. These risk factors were further evaluated using a leave-one-out analysis, systematically excluding individual studies to assess their impact on the overall results. Notably, after performing the leave-one-out analysis, the associations for hemoglobin (Hb) and platelet count became statistically significant (Table 3) (Fig. 10, Fig. 11).

**Table 3:** Leave-one-out analysis.

Risk factor	No. of studies	Initial I^2^	Study removed	Final I^2^	Final P value
Hb	3	76 %	Kalçık 2019	29 %	0.04
Tumor size	10	86 %	Kalçık 2019	78 %	Unchanged
BMI	3	94 %	Yin 2016	0 %	Unchanged
LAD	5	96 %	Yin 2016	37 %	Unchanged
Age	16	81 %	Yin 2016	72 %	Unchanged
LDL	3	87 %	Gao 2014	65 %	Unchanged
Platelet count	6	90 %	Canga 2017	0 %	<0.0001
Attachment size^a^	2	80 %	–	–	–

a Since there are only two studies, a leave-one-out analysis isn't feasible.

### 3.5 Publication bias

Evidence of substantial bias was found for age and irregular surface (*p* < 0.05) after performing Egger's test. This test was not performed for risk factors reported in <10 studies.

## 4 Discussion

In our meta-analysis of 28 risk factors across 18 studies, we found that New York Heart Association (NYHA) class I/II, hypertension, hyperlipidemia, coronary artery disease, high tumor mobility and irregular tumor surface were significantly associated with an increased risk of embolism in patients with cardiac myxomas. Conversely, higher mean platelet volume and female gender were associated with a reduced risk. Following a leave-one-out analysis, higher platelet count and hemoglobin levels were also linked to an increased risk of embolism. Notably, our findings on female gender, hyperlipidemia, and coronary artery disease, tumor mobility and mean platelet volume differ from previous meta-analysis. In contrast to previous meta-analysis, our study did not find a significant association between tumor location and embolism, nor between tumor base size and embolism.

Hyperlipidemia, known for its role in increasing the risk of thrombosis by promoting endothelial dysfunction, may similarly contribute to the pathogenesis of embolism in myxoma patients [[15], [16], [17]]. Our study also found that hypertension is related to increased risk of embolism. It is well known that hypertension confers hypercoagulable or prothrombotic state which can lead to increased clot formation on the tumor and subsequent embolization [18,19].

Evidence indicates a strong association between heart failure and the formation of cardiac thrombi as well as arterial embolization [20]. However, our study found that NYHA class I/II was associated with a higher risk of embolism compared to NYHA class III/IV, which aligns with findings from previous meta-analysis. This observation may be explained by the fact that patients in the embolic group were often diagnosed earlier in the disease progression, typically following an acute embolic event. At this stage, the tumor size and blockage may not be sufficiently advanced to cause symptoms, leading to classification within the lower severity group according to the NYHA system. In contrast, it is suggested that patients in the non-embolic group tend to have a more prolonged disease course, during which they are more likely to develop hemodynamic symptoms [9].

Another notable finding from our study is the lack of a significant difference in left ventricular ejection fraction between the embolic and non-embolic groups. This suggests that the heart failure symptoms observed in the non-embolic group may be attributable to obstructive heart failure caused by the myxoma, which can occur with preserved ejection fraction [21,22].

Tumor characteristics significantly associated with embolism included tumor mobility and surface characteristics. Due to the varied reporting across different studies, certain features such as gelatinous or soft consistency, papillary (villous) morphology, and polypoid appearance were categorized under the umbrella term of “irregular surface characteristics.” In contrast, smooth solid exterior, with firm compact consistency and round appearance were grouped under “regular surface characteristics,” consistent with the traditional type 1 and type 2 classification used in previous studies [23,24]. The increased incidence of embolism in tumors with irregular surface characteristics may be attributed to their high friability [21]. Additionally, these tumors tend to have a greater propensity for spontaneous fragmentation, which can be exacerbated by papillary or villous morphology due to increased secretion of metalloproteinases and other enzymes in such tumors [21,25]. Tumor mobility was also found to be associated with a higher risk of embolism, potentially due to mechanical fragmentation and thrombus formation on the tumor surface, which could contribute to embolic events. The prolapsing nature of the tumor showed an increased risk of embolism, although this was of borderline statistical significance. Contrary to prior meta-analysis, neither the base or attachment size nor tumor location (typical/atrial) was found to be associated with embolism [9].

Among hematological parameters, a higher mean platelet volume (MPV) was found to be associated with an increased risk of embolism. MPV is known to correlate with platelet activity, and activation of megakaryocytes by cytokines IL-6, IL-1, and TNF alpha [26]. Cardiac myxomas are also known to produce cytokines, which could facilitate the process of megakaryocyte activation and subsequently increase MPV [27].

Studies have previously demonstrated an association between elevated platelet counts and thromboembolic events in various settings [27,28]. Interestingly, while the cytokine IL-6, known to be associated with cardiac myxomas [27], is often correlated with lower hemoglobin levels in the context of COVID-19 [29], no substantial data exist on its direct impact on embolism in myxoma patients. In our study, both platelet count and hemoglobin levels were found to be significantly associated with embolic events following a leave-one-out analysis. However, further research is required to better establish these relationships and clarify the underlying mechanisms.

### 4.1 Limitations

This meta-analysis is an updated review on this topic, incorporating additional studies not covered in previous analyses. There are notable limitations inherent in this review. First, data from certain studies included in the previous analysis exclusively available in Chinese databases could not be accessed, potentially limiting the comprehensiveness of the analysis. Second, all included studies are retrospective and observational in nature, which introduces inherent design biases that cannot be fully mitigated. Third, several studies within this meta-analysis reported sample sizes smaller than 50, which may compromise the statistical power of the pooled estimates. Fourth, significant heterogeneity was identified among studies for multiple risk factors, highlighting the need for future high-powered studies to provide more clarity. These limitations should be considered when interpreting the results of this meta-analysis.

## CrediT authorship contribution statement

**Muhammad Ahmad Qureshi:** Formal analysis, Conceptualization. **Danyal Bakht:** Writing – review & editing, Writing – original draft, Formal analysis. **Omair Ahmed:** Writing – review & editing, Writing – original draft. **Shahan Haseeb:** Writing – review & editing. **Kartik Gupta:** Writing – review & editing. **Omar Baqal:** Writing – review & editing, Conceptualization. **Maaz Amir:** Data curation. **Khawar Ali:** Data curation. **Mirza Muhammad Hadeed Khawar:** Data curation. **Muqaddas Hussain:** Writing – review & editing. **Luqman Munir:** Writing – review & editing. **Hussein Othman:** Writing – review & editing.

## Ethics approval

This study did not require an IRB approval.

## Disclosure statement

The authors have nothing to disclose.

## Word count

Meeting findings were presented at: American Heart Association Scientific Sessions 2024, Moderated Digital Poster, Chicago, IL, USA, November 16–18, 2024.

## Declaration of Generative AI and AI-assisted technologies in the writing process

AI (Chat GPT 3.5) was used for error removal from the final draft only. Authors take full responsibility for the content.

## Source of funding

The study did not require any funding.

## Declaration of competing interest

The authors declare that they have no known competing financial interests or personal relationships that could have appeared to influence the work reported in this paper.

## Data Availability

All data and materials used in the manuscript are from already published resources.

## Appendix A Supplementary data

Supplementary material 1 Image 1

Supplementary material 2 Image 2
