# Supplementary material for: Evaluating risk factors of embolism in patients with cardiac myxoma: A systematic review and meta-analysis
Source: Am Heart J Plus. 2025 May 29;56:100559. doi: 10.1016/j.ahjo.2025.100559 (PMC12182336; doi:10.1016/j.ahjo.2025.100559)
Supplement: Supplementary file 1 — Supplementary material 1 [file mmc1.docx]

|  |  |  |  | Sample size | | Age (Mean ±SD or median(IQR)) | |  |  |
| --- | --- | --- | --- | --- | --- | --- | --- | --- | --- |
| Author name | **Year of Study** | **Country of Study** | **Type of study** | **Embolism** | **Non embolism** | **Embolism group** | **Non embolism group** | **Incidence of embolism** | **Statistically significant Risk Factor** |
| Alvarez-Sabín et al. (1) | 2001 | Spain | Retrospective study | 11 | 17 | 45±15 | 58±13 | 39.29% | Female gender |
| Boyacıoğlu et al. (2) | 2017 | Turkey | Retrospective study | 25 | 74 | 49±16.89 | 50.12±15.71 | 25.25% | Tumor Surface, NYHA. |
| Canga et al. (3) | 2017 | Turkey | Retrospective study | 13 | 53 | 51.1±11.4 | 55.9±12.4 | 19.70% | Female gender, irregular surface, LDL, |
| Gabe ED et al. (4) | 2002 | Argentina | Retrospective study | 13 | 18 | 53.38±20 | 54.33±17 | 41.94% | - |
| Ha et al. (5) | 1999 | Korea | Retrospective study | 7 | 18 | 46±17 | 59±10 | 28.00% | Tumor Surface |
| He et al. (6) | 2015 | China | Retrospective study | 33 | 129 | 48 (38–61) | 54 (46–63) | 20.37% | Tumor location, Tumor surface, MPV |
| Wen et al. (7) | 2018 | China | Retrospective study | 17 | 113 | 39.9±12.6 (13‑78) | 39.9±12.6 (13‑78) | 13.07% | Tumor Surface |
| Kalçık et al. (8) | 2019 | Turkey | Retrospective study | 13 | 80 | 50 (36–62) | 56 (45–65) | 13.98% | Tumor surface |
| Lee et al. (9) | 2012 | Korea | Retrospective study | 13 | 46 | 59.2±12.6 | 58.3±12.6 | 22.03% | Tumor surface |
| Gao et al. (10) | 2014 | China | Retrospective study | 15 | 12 | 56.4±13.3 | 58.4±14.1 | 55.56% | Tumor surface |
| Ma et al. (11) | 2023 | China | Retrospective study | 61 | 355 | 56.17±13.10 | 53.41±13.97 | 14.66% | Hypertension, Hyperlipidemia, Mobility |
| Qiao et al. (12) | 2023 | China | Retrospective study | 54 | 348 | 55.7±12.2 | 54.3±13.6 | 13.43% | Tumor surface, Female gender, Hyperlipidemia, CAD, Mobility |
| Wang et al. (13) | 2016 | China | Retrospective study | 32 | 175 | 39.7±16.6 | 45.0±15.5 | 15.46% | Tumor Surface |
| Yin et al. (14) | 2016 | China | Retrospective study | 143 | 322 | 59.4±10.9 | 52.3±12.4 | 30.75% | Hypertension, , CAD, Tumor surface, |
| Zhang et al. (15) | 2021 | China | Retrospective study | 23 | 137 | 60.48±9.69 | 55.93±13.27 | 14.38% | Hyperlipidemia, CAD |
| Zheng et al. (16) | 2014 | China | Retrospective study | 15 | 63 | 49±9 | 52±6 | 19.23% | Tumor Surface |
| Amemiya et al. (17) | 2022 | Japan | Retrospective study | 23 | 83 | 60±14 | 60±14 | 21.70% | - |
| Brinjikji et al. (18) | 2015 | USA | Retrospective study | 14 | 33 | 63.2±17.4 | 63.2±17.4 | 29.79% | - |

**Table 1: Characteristics of included studies**

**References:**

1. Alvarez-Sabín J, Lozano M, Sastre-Garriga J, Montoyo J, Murtra M, Abilleira S, et al. Transient ischaemic attack: a common initial manifestation of cardiac myxomas. Eur Neurol. 2001;45(3):165–70.

2. Boyacıoğlu K, Kalender M, Dönmez AA, Çayhan B, Tuncer MA. Outcomes following embolization in patients with cardiac myxoma. Journal of Cardiac Surgery. 2017;32(10):621–6.

3. Çanga Y, Karataş MB, Çalık AN, Bezgin T, Tanık VO, Yıldız U, et al. Cardiac Myxoma: Fourteen-Year Experience of a Tertiary Reference Center. Kosuyolu Heart Journal. 2017 Dec 20;20(3):210–6.

4. Gabe ED, Rodríguez Correa C, Vigliano C, San Martino J, Wisner JN, González P, et al. Cardiac myxoma. Clinical-pathological correlation. Revista Espanola de Cardiologia. 2002;55(5):505–13.

5. Ha JW, Kang WC, Chung N, Chang BC, Rim SJ, Kwon JW, et al. Echocardiographic and morphologic characteristics of left atrial myxoma and their relation to systemic embolism. American Journal of Cardiology. 1999 Jun 1;83(11):1579–82.

6. He DK, Zhang YF, Liang Y, Ye SX, Wang C, Kang B, et al. Risk factors for embolism in cardiac myxoma: a retrospective analysis. Med Sci Monit. 2015;21:1146–54.

7. Wen XY, Chen YM, Yu LL, Wang SR, Zheng HB, Chen ZB, et al. Neurological manifestations of atrial myxoma: A retrospective analysis. Oncol Lett. 2018 Oct;16(4):4635–9.

8. Kalçık M, Bayam E, Güner A, Küp A, Kalkan S, Yesin M, et al. Evaluation of the potential predictors of embolism in patients with left atrial myxoma. Echocardiography. 2019 May;36(5):837–43.

9. Lee SJ, Kim JH, Na CY, Oh SS. Eleven years’ experience with Korean cardiac myxoma patients: focus on embolic complications. Cerebrovasc Dis. 2012;33(5):471–9.

10. Long Y, Gao C. Brain embolism secondary to cardiac myxoma in fifteen Chinese patients. ScientificWorldJournal. 2014;2014:718246–718246.

11. Ma L, Cai B, Qiao ML, Fan ZX, Fang LB, Wang CB, et al. Risk factors assessment and a Bayesian network model for predicting ischemic stroke in patients with cardiac myxoma. Front Cardiovasc Med. 2023;10:1128022.

12. Qiao ML, Ma L, Wang CB, Fang LB, Fan ZX, Niu TT, et al. Clinical features, risk factors and survival in cardiac myxoma-related ischemic stroke: A multicenter case-control study. J Neurol Sci. 2023 Jan 15;444:120517.

13. Wang Z, Chen S, Zhu M, Zhang W, Zhang H, Li H, et al. Risk prediction for emboli and recurrence of primary cardiac myxomas after resection. J Cardiothorac Surg. 2016;11:22–22.

14. Yin L, Wang J, Li W, Ling X, Xue Q, Zhang Y, et al. Usefulness of CHA2DS2-VASc Scoring Systems for Predicting Risk of Perioperative Embolism in Patients of Cardiac Myxomas Underwent Surgical Treatment. Scientific reports. 2016;6:39323–39323.

15. Zhang Y, Ye Z, Fu Y, Zhang Z, Ye Q, Chen F, et al. Characterizations of ischemic stroke complications in cardiac myxoma patients at a single institution in Eastern China. Neuropsychiatric Disease and Treatment. 2021;17:33–40.

16. Zheng Z, Guo G, Xu L, Lei L, Wei X, Pan Y. Left atrial myxoma with versus without cerebral embolism: length of symptoms, morphologic characteristics, and outcomes. Tex Heart Inst J. 2014;41(6):592–5.

17. Amemiya K, Yonemoto Y, Ishibashi-Ueda H, Matsumoto M, Ohta-Ogo K, Ikeda Y, et al. Morphological characteristics of cardiac myxoma causing embolism: a series of 40 years of experience at a single institute. Virchows Archiv. 2023;482(2):377–84.

18. Brinjikji W, Morris JM, Brown RD, Thielen KR, Wald JT, Giannini C, et al. Neuroimaging Findings in Cardiac Myxoma Patients: A Single-Center Case Series of 47 Patients. Cerebrovasc Dis. 2015;40(1):35–44.

19. Kalçık M, Bayam E, Güner A, Küp A, Kalkan S, Yesin M, et al. Evaluation of the potential predictors of embolism in patients with left atrial myxoma. Echocardiography. 2019;36(5):837–43.

20. Ma L, Cai B, Qiao ML, Fan ZX, Fang LB, Wang CB, et al. Risk factors assessment and a Bayesian network model for predicting ischemic stroke in patients with cardiac myxoma. Frontiers in Cardiovascular Medicine [Internet]. 2023;10. Available from: ["https://www.embase.com/search/results?subaction=viewrecord&id=L2022435640&from=export", "http://dx.doi.org/10.3389/fcvm.2023.1128022"]

21. Çanga Y, Karataş MB, Çalık AN, Bezgin T, Tanık VO, Yıldız U, et al. OP-094 [AJC » Cardiac imaging - Echocardiography] Cardiac Myxoma: Fourteen-year experience of a Tertiary Reference Center. American Journal of Cardiology. 2017 Apr 15;119(8):e5–6.

22. Neurological manifestations of atrial myxoma: A retrospective analysis [Internet]. [cited 2024 Aug 6]. Available from: https://www.spandidos-publications.com/ol/16/4/4635

23. Qiao ML, Ma L, Wang CB, Fang LB, Fan ZX, Niu TT, et al. Clinical features, risk factors and survival in cardiac myxoma-related ischemic stroke: A multicenter case-control study. Journal of the Neurological Sciences [Internet]. 2023;444. Available from: ["https://www.embase.com/search/results?subaction=viewrecord&id=L2021795667&from=export", "http://dx.doi.org/10.1016/j.jns.2022.120517"]

**1**

**Tumor surface characteristics classification adopted for our study**

Irregular: Gelatinous, gelatinous exterior, villous type, soft texture, soft consistency, papillary consistency, polypoid type, atypical type = type 1 pathology

Regular: Encapsulated, solid exterior, smooth surface, firm texture, compact consistency, solid consistency, round type, typical type = type 2 pathology

**Study-wise classification**

**Amemiya 2022**

Irregular + Villous vs Smooth.

The smooth type specimens had a round shape, with complete or incomplete capsulation and solid appearance. The irregular type specimens did not have a capsulated or smooth

shape and soft external appearance. The villous type specimens had a gelatinous external appearance with a papillary or frond-like villi appearance

**Boyacıoğlu 2017**

Papillary vs solid

Tumors with a smooth regular border and a solid consistency were classified as solid; papillary myxomas were characterized by an irregular and gelatinous exterior with friable, soft consistency.

**Canga 2017**

**Type 1 vs type 2**

**Myxomas were examined in two groups, Type 1 and Type 2, according to the macroscopic surface characteristics in the pathology results**. Soft-textured myxomas with an irregular or villous surface were classified as Type 1, while those with a smooth surface and firmer texture were classified as Type 2.

**Gao 2014**

Irregular vs regular shape/morphology

**Ha 1999**

Polypoid vs Round

Two distinct types of myxoma could be identified: round type, characterized by solid and round shape with nonmobile surface (n 5 13, 52%) (Figure 1); and polypoid type, characterized by soft and irregular shape with mobile surface

**He 2015**

Irregular vs regular surface

**Kalçık 2019**

Irregular or villous vs Regular or smooth

Tumors with regular borders were classified as smooth, which was characterized by a round shape and a solid surface. The others which were characterized by a soft and irregular shape and a gelatinous surface with cauliflower appearance were classified as villous.

**Lee 2012**

Irregular vs regular

The 59 myxomas were divided into two types according to the criteria described in previous clinicopathologic studies: type 1, with an irregular or villous surface and a soft consistency, and type 2, with a smooth surface and a compact consistency.

**Ma 2023**

Irregular vs regular

**Qiao 2023**

Irregular vs regular

**Wang 2016**

Gelatinous vs encapsulated

CMs were divided into solid and encapsulated or soft and gelatinous based on the classification system by Ha et al. Tumors with smooth regular borders were classified as solid, which was characterized by a round shape and a non-mobile surface. The gelatinous CMs were characterized by a soft and irregular shape and a mobile surface. The gelatinous tumors were more friable, often with a cauliflower appearance necessitating piecemeal removal.

**Wen 2018**

Regular vs irregular

**Yin 2016**

Typical vs atypical

Atypical type of myxomas according to previous report (myxomas with soft consistency and very fine villous extensions, an irregular or villous surface)

**Zheng 2014**

Type 1 vs type 2

The myxomas were divided into 2 types, in accordance with the criteria described in previous clinicopathologic studies: type 1, with an irregular or villous surface and a soft consistency; and type 2, with a smooth surface and a compact consistency.

2

**Search Strategy**

**PubMed:** 906 results

((("Heart"[Mesh] OR "Heart Neoplasms"[Mesh] OR heart*[tw] OR cardiac*[tw]) AND ("Myxoma"[Mesh] OR "Carney Complex"[Mesh] OR "Atrial myxoma, familial" [Supplementary Concept] OR Myxoma*[tw] OR "Atrial Myxoma*"[tw] OR angiomyxoma*[tw])) AND ("Embolism"[Mesh] OR "Intracranial Embolism"[Mesh] OR "Embolism, Paradoxical"[Mesh] OR "Embolism and Thrombosis"[Mesh] OR "Pulmonary Embolism"[Mesh] OR "Intracranial Embolism and Thrombosis"[Mesh] OR "Ischemic Stroke"[Mesh] OR embolus*[tw] OR embolism*[tw] OR thrombus*[tw])) AND ("Risk Factors"[Mesh] OR "Causality"[Mesh] OR "etiology" [Subheading] OR associat*[tw] OR determinant*[tw] OR etiology*[tw] OR "risk factor*"[tw] OR predict*[tw])

**Embase:** 980 results

(('risk factor*' OR associat* OR predict* OR determinant* OR etiology*) AND (embolus* OR embolism* OR thrombus*) AND (myxoma* OR angiomyxoma* OR 'atrial myxoma*') AND (cardiac OR heart))/br

**Cochrane:** 5 results

#1: embolus OR embolism

#2: heart OR cardiac

#3: myxoma OR angiomyxoma

#4: #1 AND #2 AND #3

3

**Risk of Bias Assessment of included studies using Newcastle Ottawa Scale**

| **Study** | **Selection** | | | | **Comparability** | **Outcome** | | | **Total** |
| --- | --- | --- | --- | --- | --- | --- | --- | --- | --- |
|  | 1 | 2 | 3 | 4 | 1 | 1 | 2 | 3 |  |
| **Gabe 2002 (4)** | ★ | ★ | ★ |  | ★★ | ★ | ★ | ★ | 8 |
| **Zhang 2021** (15) | ★ | ★ | ★ |  | ★★ | ★ | ★ |  | 7 |
| **Lee 2012** (9) | ★ | ★ | ★ |  | ★★ | ★ | ★ |  | 7 |
| **Kalçık 2019** (19) | ★ | ★ | ★ |  | ★★ | ★ | ★ | ★ | 8 |
| **Zheng 2014** (16) | ★ | ★ | ★ |  | ★★ | ★ | ★ | ★ | 8 |
| **Brinjikji 2015** (18) | ★ | ★ | ★ |  | ★ | ★ | ★ | ★ | 7 |
| **Boyacıoğlu 2017** (2) | ★ | ★ | ★ |  | ★★ | ★ | ★ | ★ | 8 |
| **Ma 2023** (20) | ★ | ★ | ★ |  | ★★ | ★ | ★ |  | 7 |
| **Canga 2017** (21) | ★ | ★ | ★ |  | ★★ | ★ | ★ | ★ | 8 |
| **Wen 2018** (22) | ★ | ★ | ★ |  | ★ | ★ | ★ | ★ | 7 |
| **Wang 2016** (13) | ★ | ★ | ★ |  | ★★ | ★ | ★ |  | 7 |
| **He 2015** (6) | ★ | ★ | ★ |  | ★★ | ★ | ★ | ★ | 8 |
| **Alvarez-Sabín 2000** (1) | ★ | ★ | ★ |  | ★ | ★ | ★ | ★ | 7 |
| **Yin 2016** (14) | ★ | ★ | ★ |  | ★★ | ★ | ★ |  | 7 |
| **Qiao 2022** (23) | ★ | ★ | ★ |  | ★★ | ★ | ★ | ★ | 8 |
| **Ha 1999** (5) |  | ★ | ★ |  | ★★ | ★ | ★ | ★ | 7 |
| **Gao 2014** (10) | ★ | ★ | ★ |  | ★★ | ★ | ★ | ★ | 8 |
| **Amemiya 2022** (17) | ★ | ★ | ★ |  | ★★ | ★ | ★ | ★ | 8 |

**Table 3: Risk of Bias Assessment Utilizing the Newcastle-Ottawa Scale**

**4**

**Funnel Plots for significant variables found in the study**

**
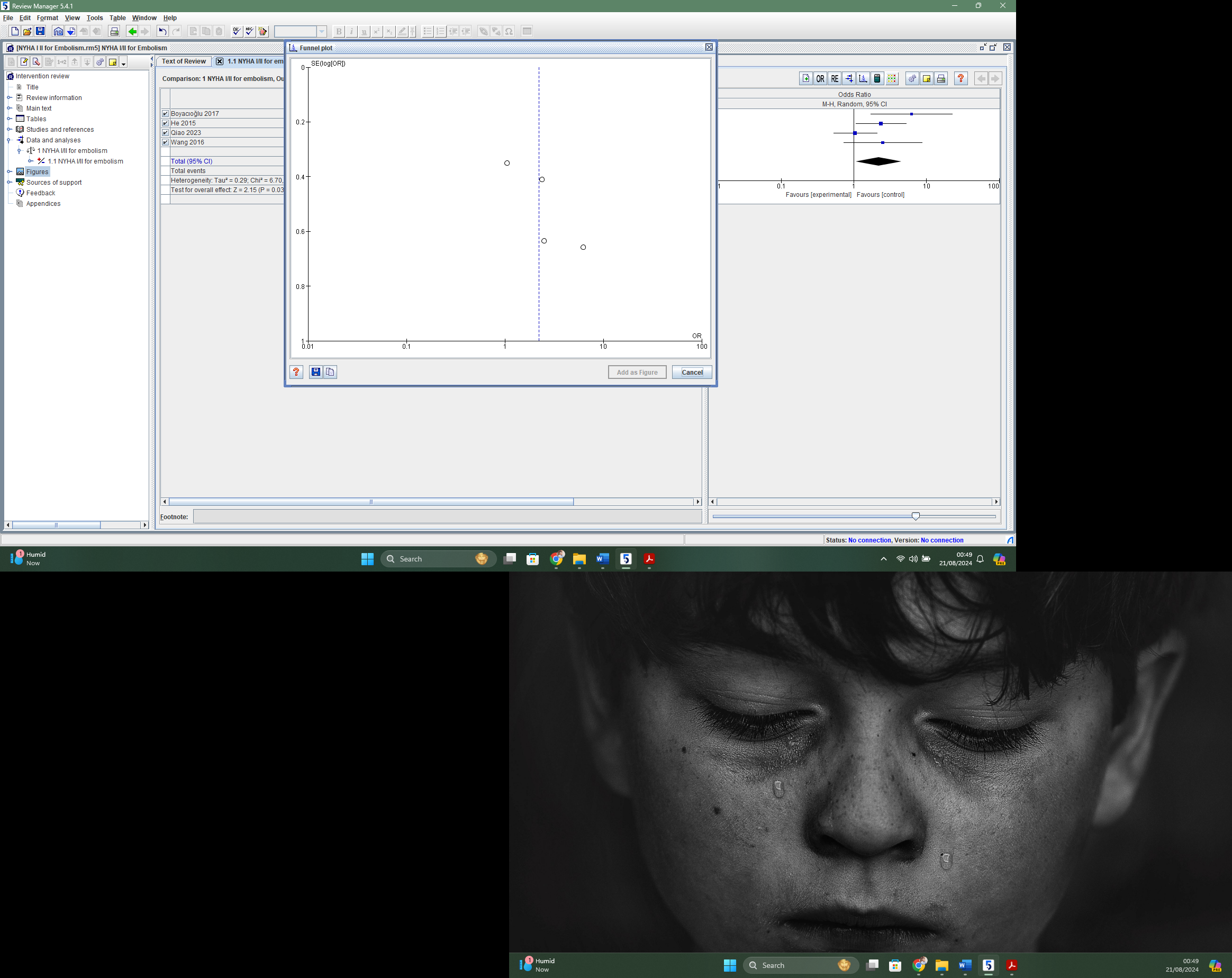
**

**Funnel plot for NYHA I/II between embolism and nonembolism**

**
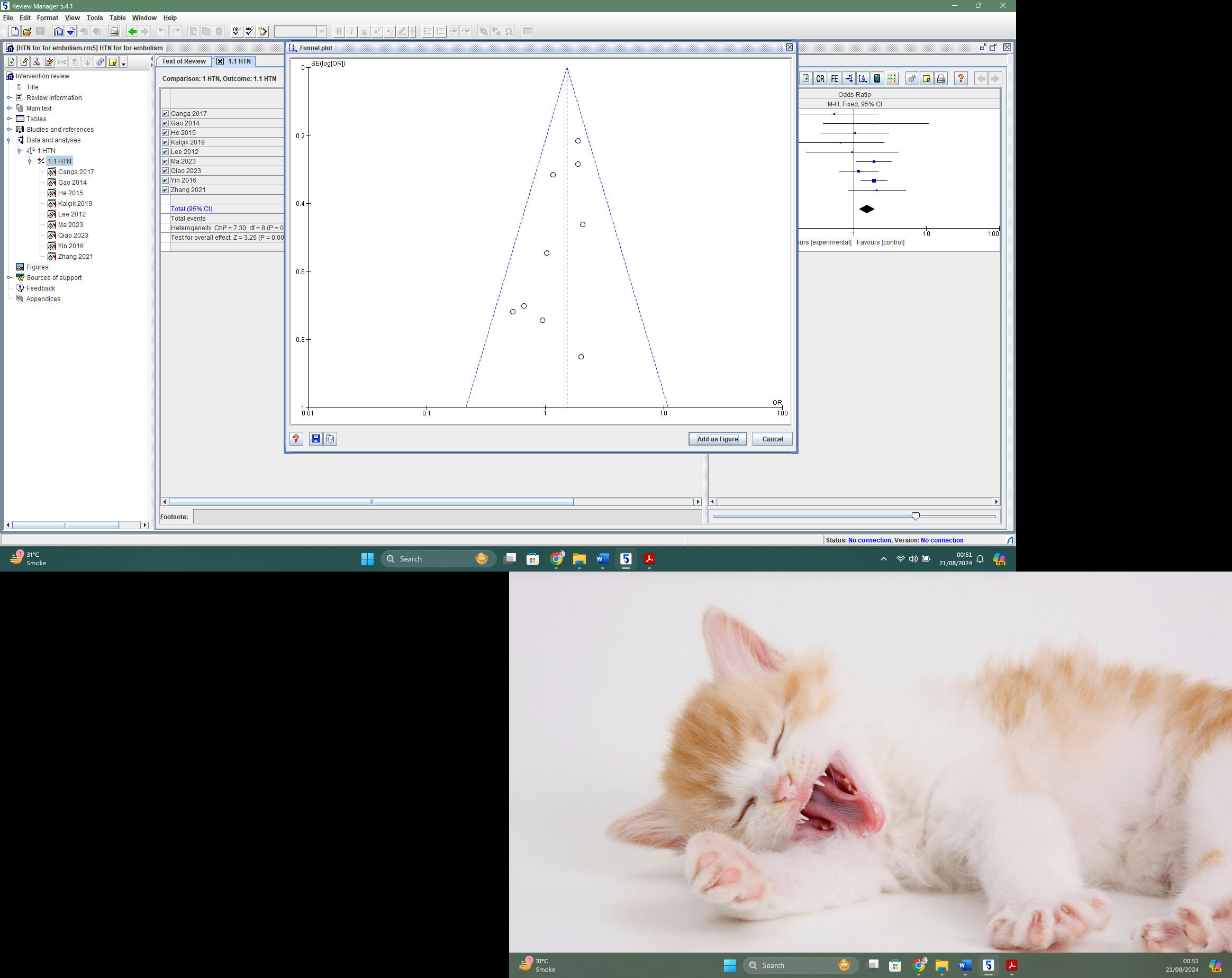
**

**Funnel plot for hypertension between embolism and nonembolism.**


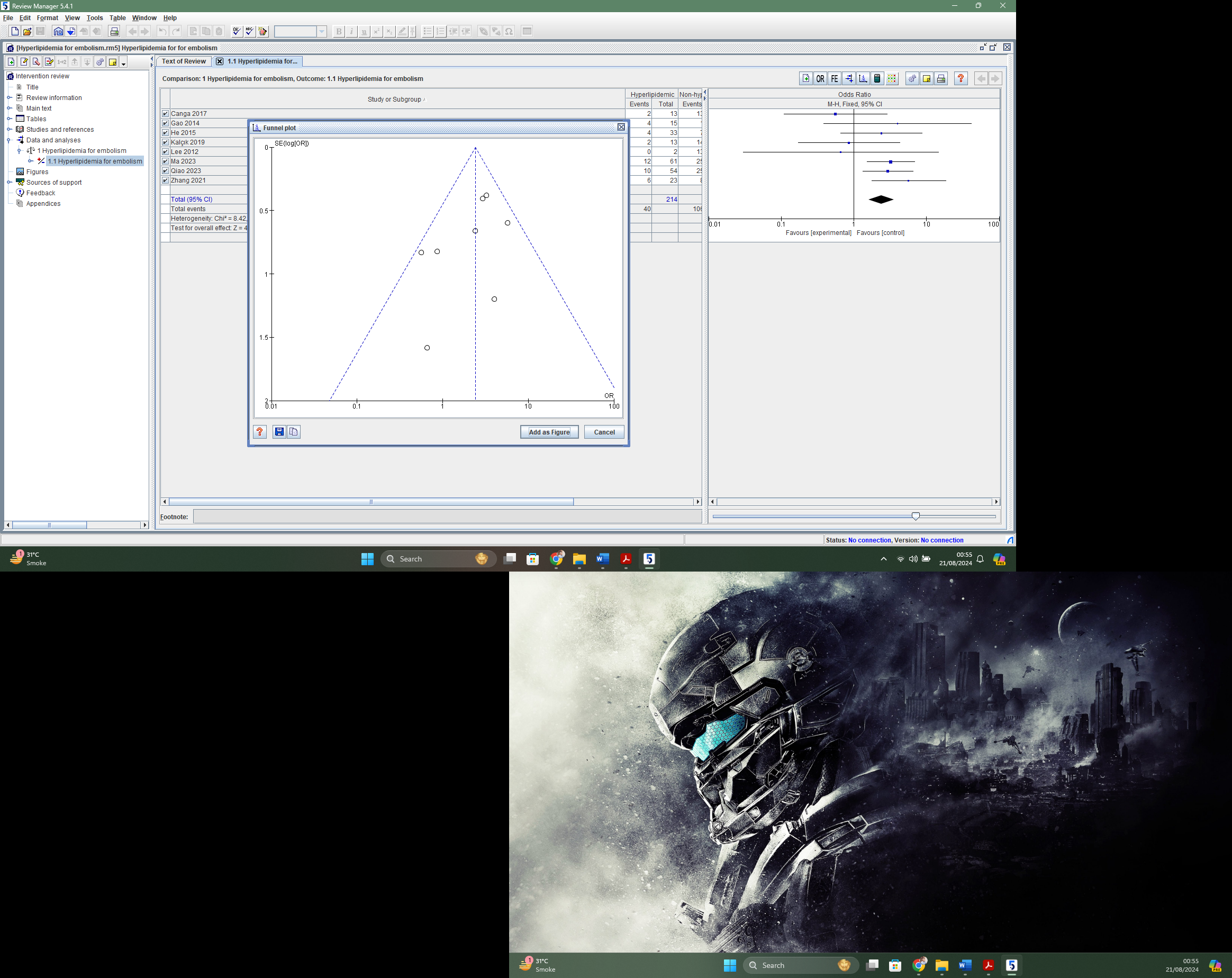


**Funnel plot for hyperlipidemia between embolism and nonembolism.**

**
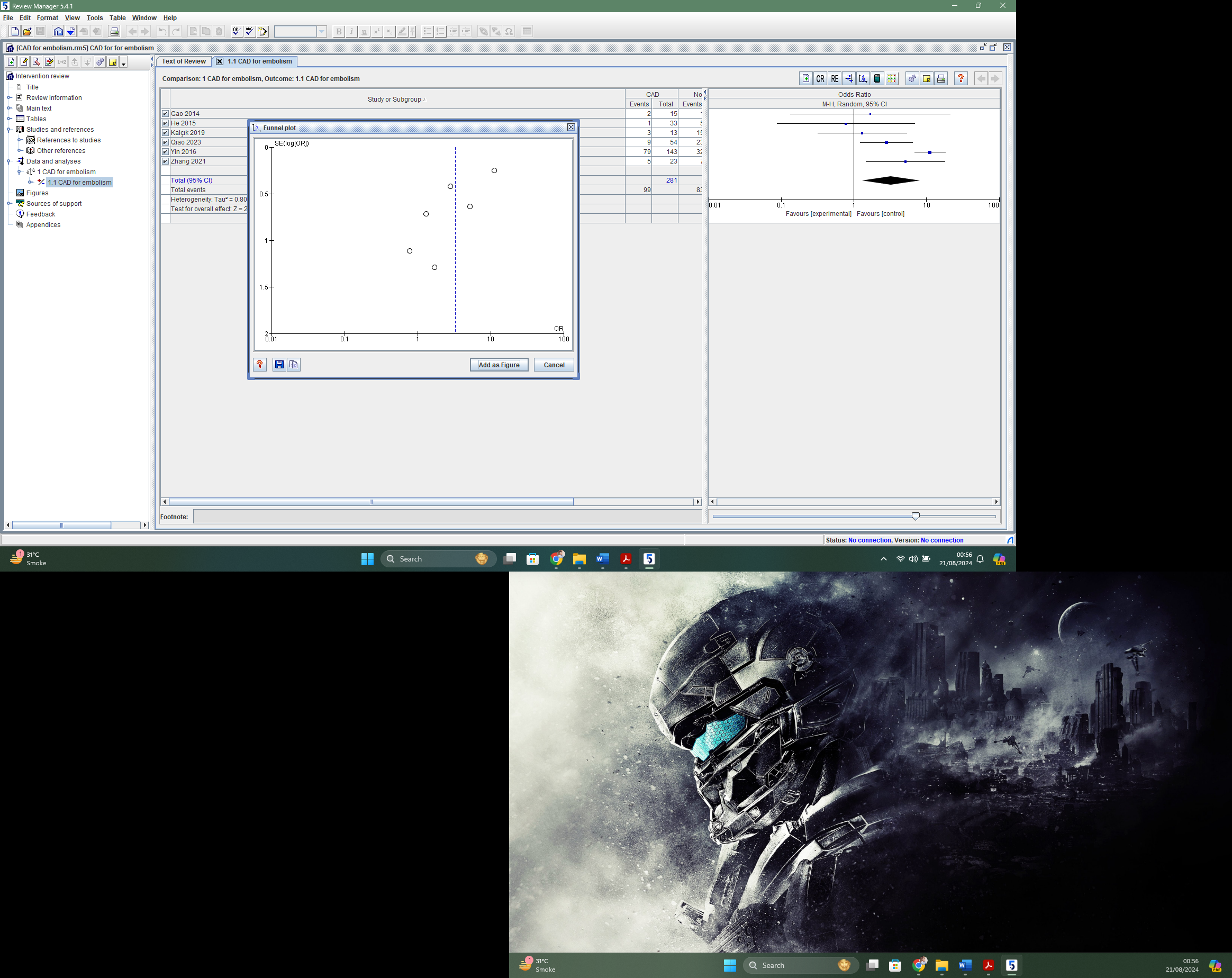
**

**Funnel plot for CAD between embolism and nonembolism.**

**
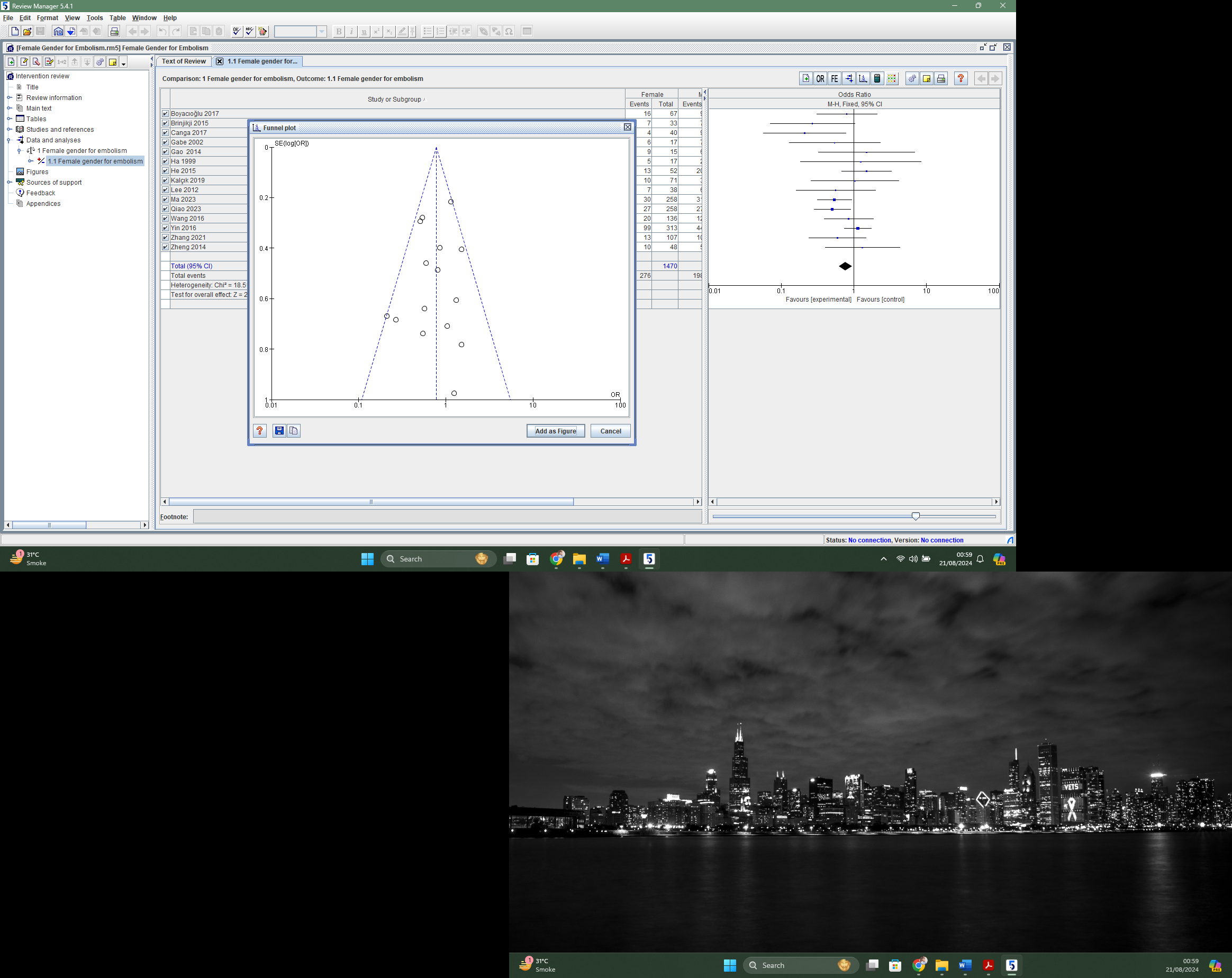
**

**Funnel plot for female gender between embolism and nonembolism.**

**
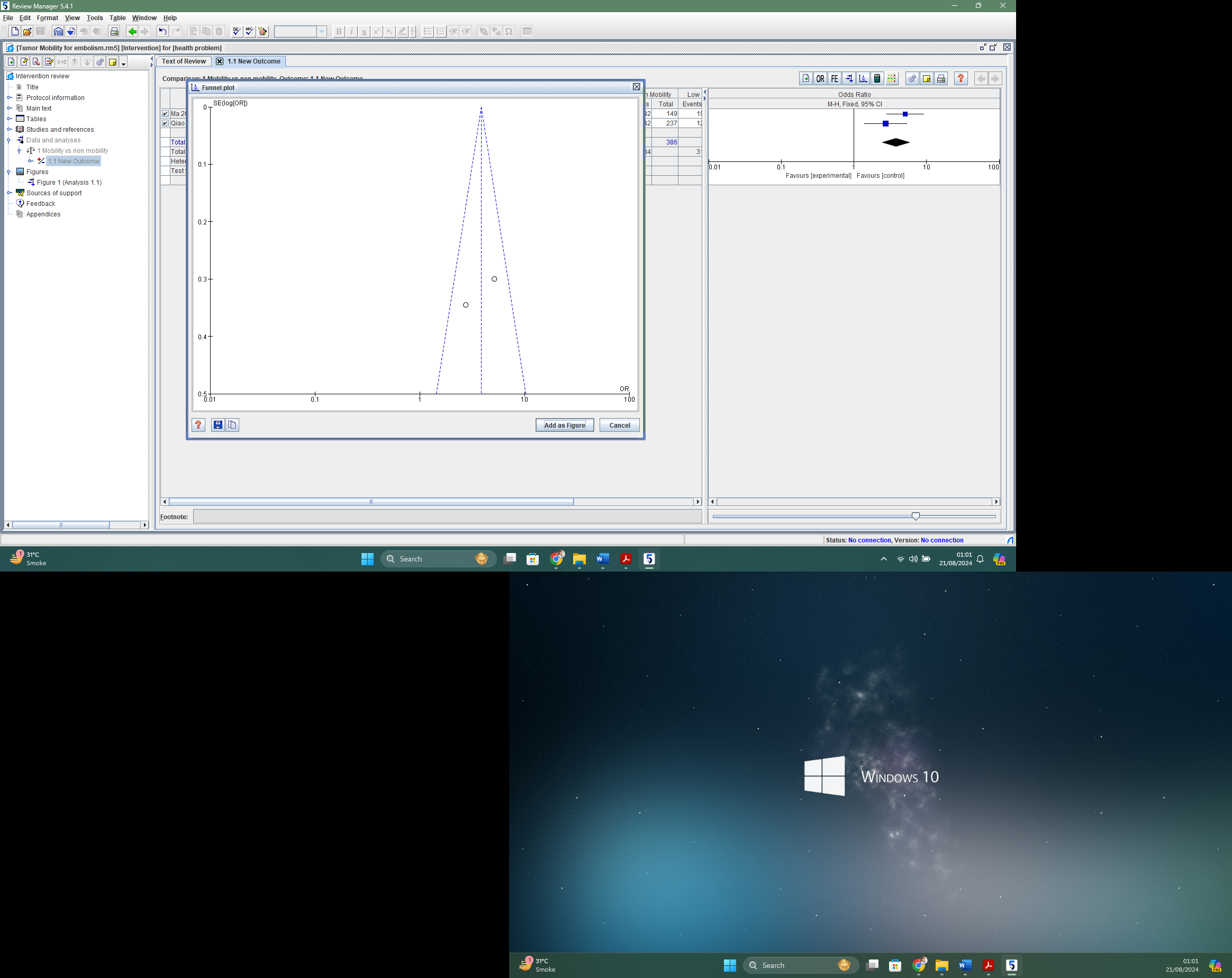
**

**Funnel plot for tumor mobility between embolism and nonembolism.**

**
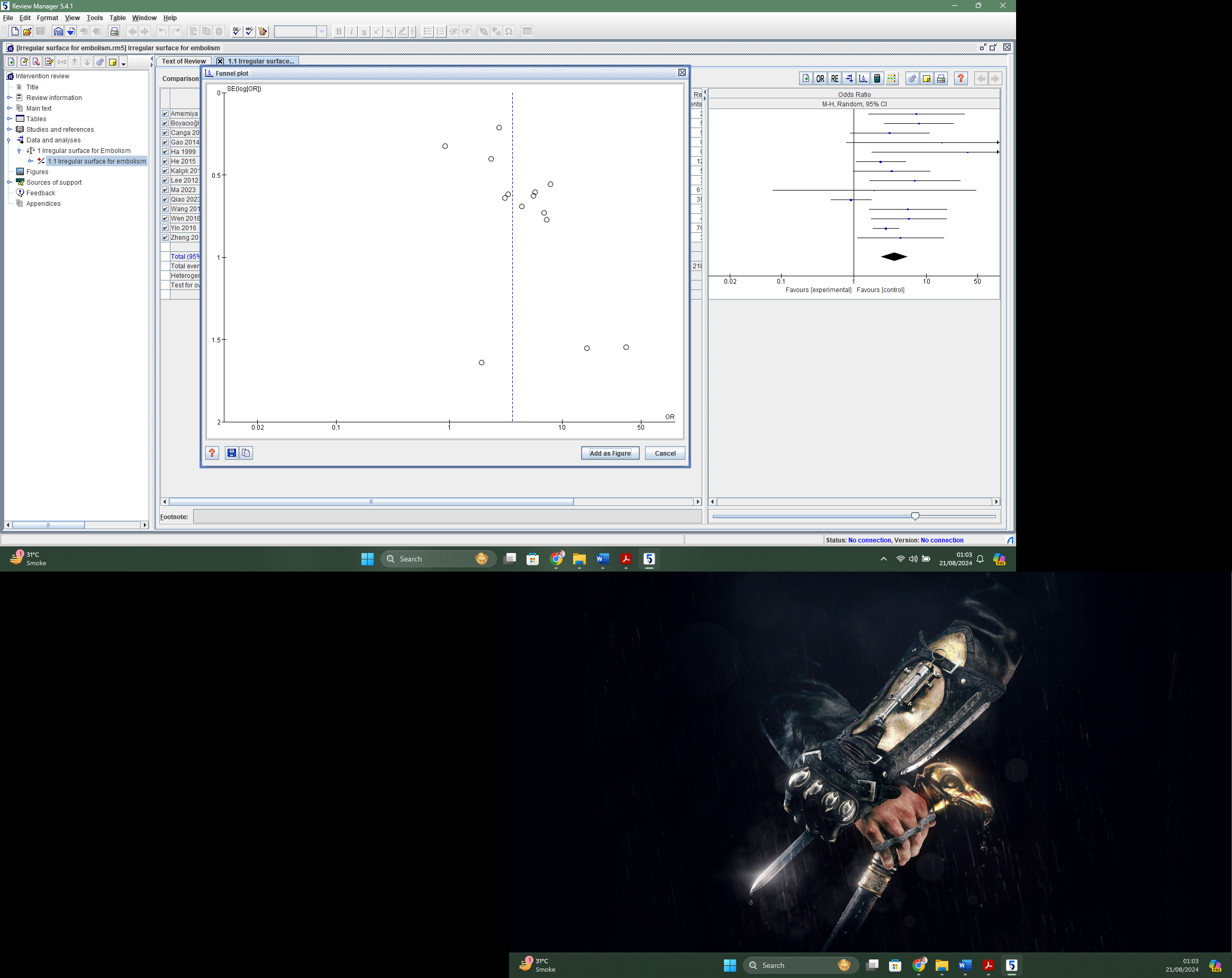
**

**Funnel plot for irregular tumor surface between embolism and nonembolism.**

**
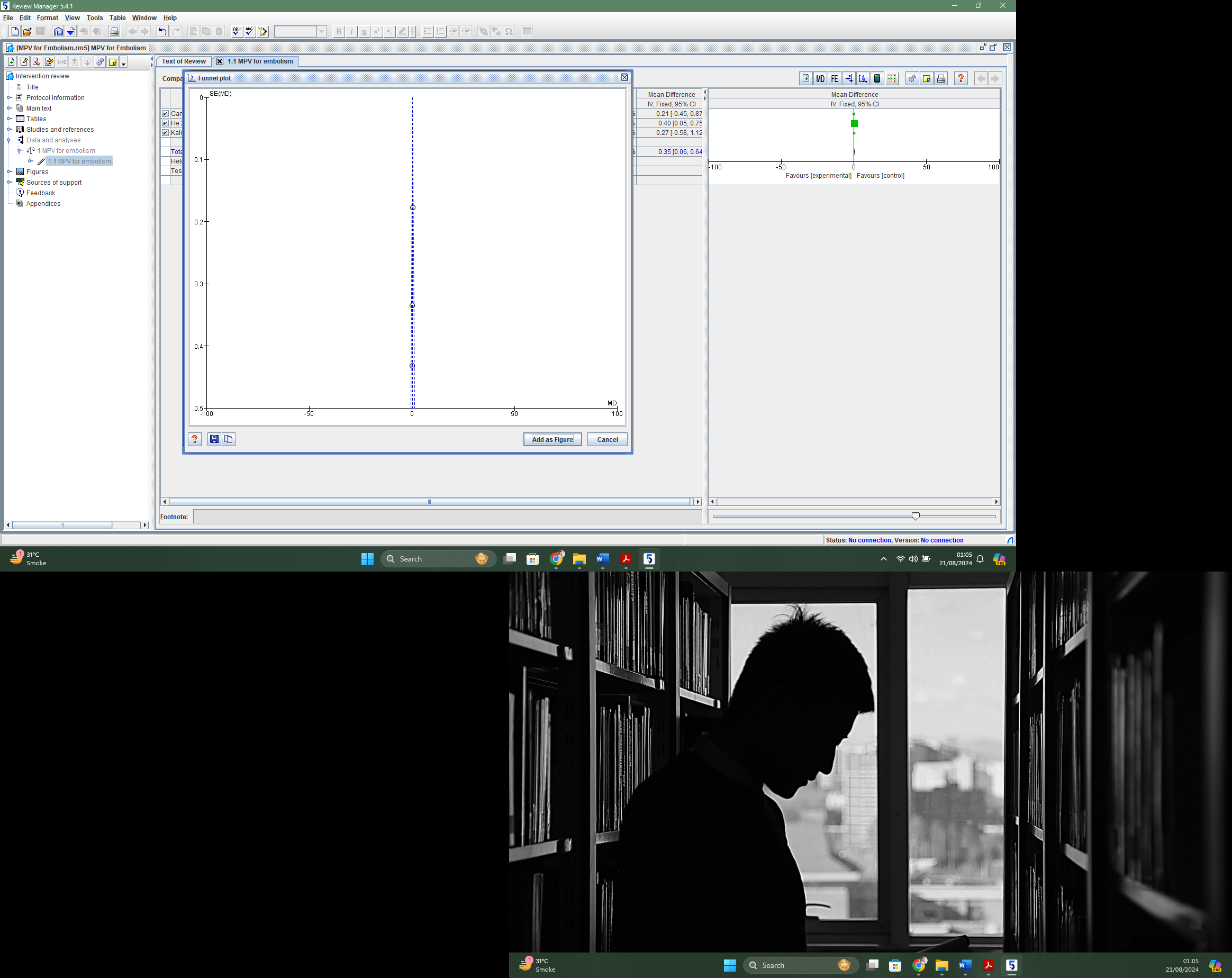
**

**Funnel plot for MPV between embolism and nonembolism.**

**5**

**Egger’s test for risk factors reported in 10 or more studies**

Gender

Model: weighted regression with multiplicative dispersion

Predictor: standard error

Test for Funnel Plot Asymmetry: t = 1.5870, df = 13, p = 0.1365

Limit Estimate (as sei -> 0): b = -1.0386 (CI: -1.5537, -0.5235)

Irregular surface

Model: weighted regression with multiplicative dispersion

Predictor: standard error

Test for Funnel Plot Asymmetry: t = 2.2854, df = 12, p = 0.0413

Limit Estimate (as sei -> 0): b = 0.0495 (CI: -0.2836, 0.3825)

Age

Model: mixed-effects meta-regression model

Predictor: standard error

Test for Funnel Plot Asymmetry: z = -2.0797, p = 0.0375

Limit Estimate (as sei -> 0): b = 2.1470 (CI: -2.5827, 6.8766)

Tumor size

Model: mixed-effects meta-regression model

Predictor: standard error

Test for Funnel Plot Asymmetry: z = 0.3237, p = 0.7462

Limit Estimate (as sei -> 0): b = -0.3010 (CI: -1.8684, 1.2664)

6

**Forrest Plots of non-significant variables**


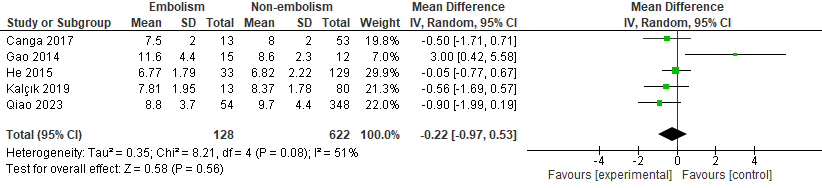


White Blood cell Count between embolism vs nonembolism


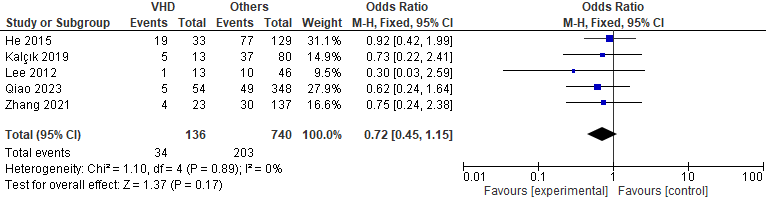


Valvular Heart disease between embolism and nonembolism


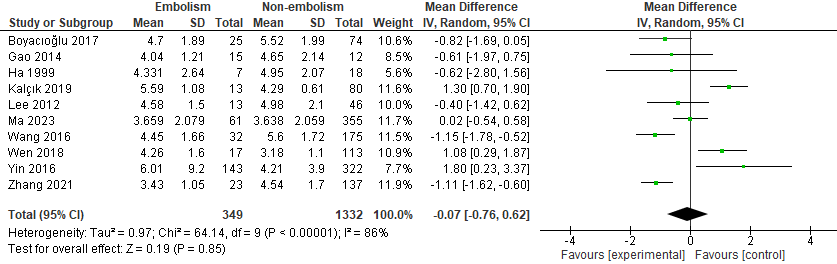


Tumor size between embolism and nonembolism


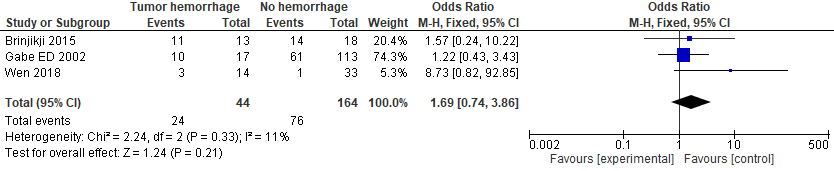


Tumor hemorrhage between embolism and nonembolism


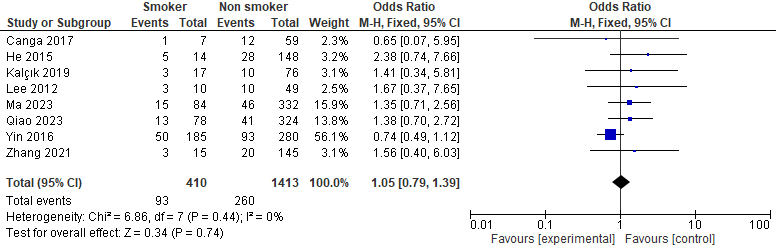


Smoking between embolism and nonembolism


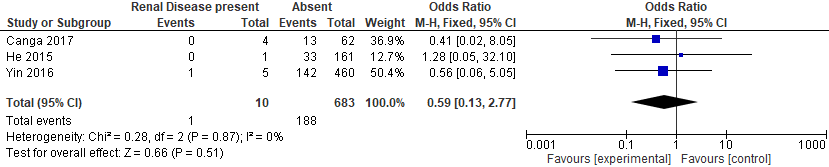


Renal disease between embolism and nonembolism


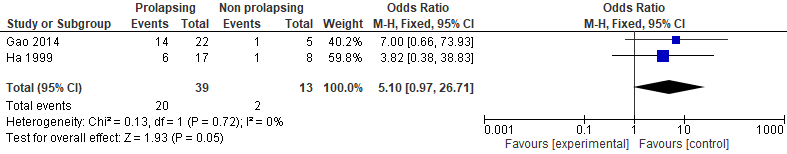


Tumor prolapse between embolism and nonembolism


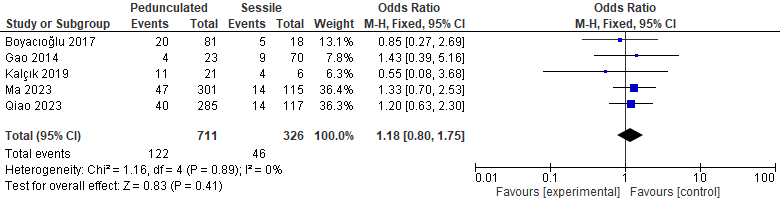


Pedunculated Appearance between embolism and nonembolism


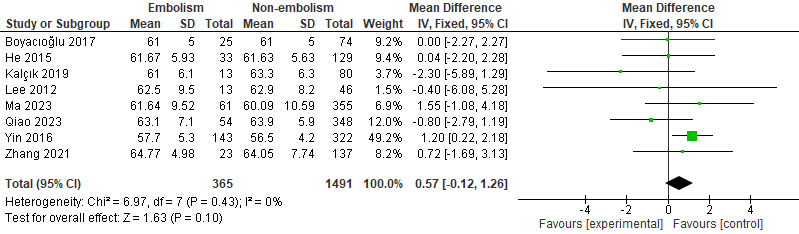


LVEF between embolism and nonembolism


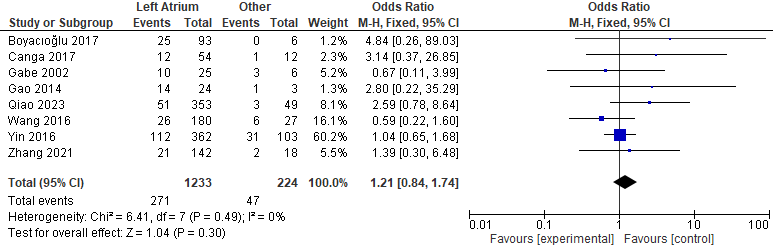


Left Atrial location between embolism and nonembolism


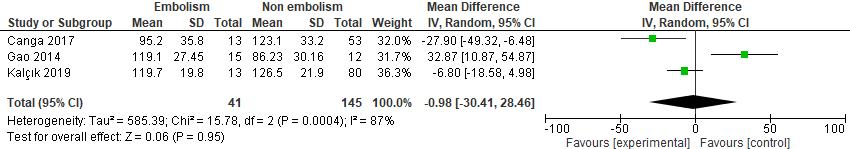


Left Atrial Dilation between embolism and nonembolism


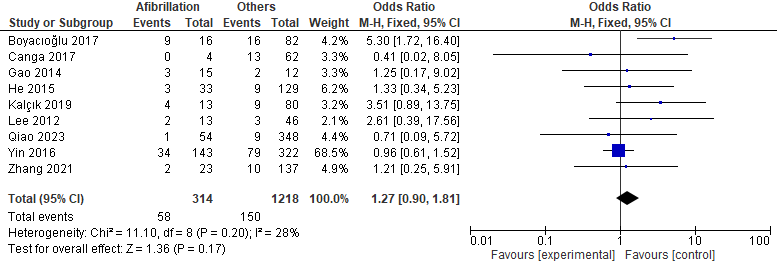


Atrial Fibrillation between embolism and nonembolism


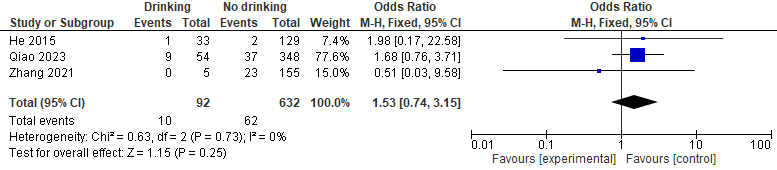


Alcohol use between embolism and nonembolism


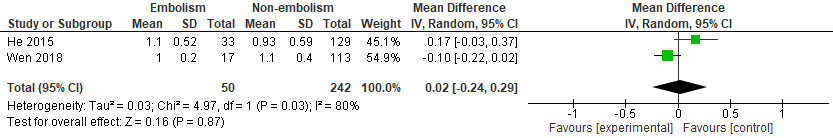


Attachment size between embolism and nonembolism


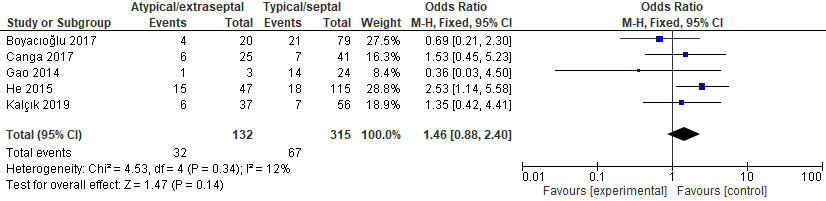


Atypical Location between embolism and nonembolism


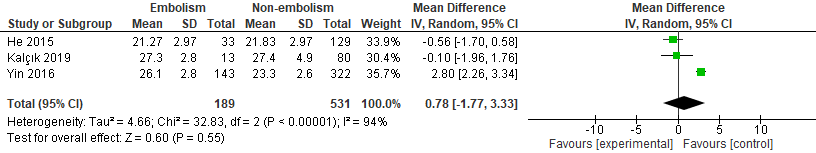


BMI between embolism and nonembolism


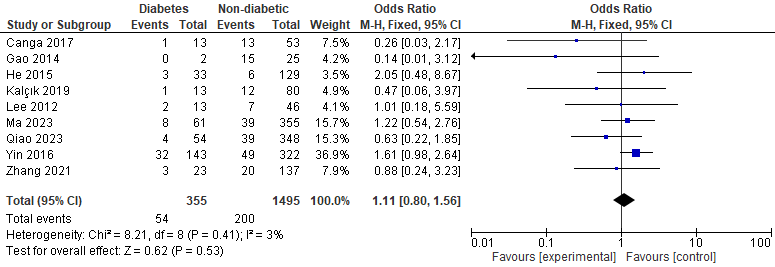


Diabetes between embolism and nonembolism
