# Supplementary material for: Evaluating risk factors of embolism in patients with cardiac myxoma: A systematic review and meta-analysis
Source: Am Heart J Plus. 2025 May 29;56:100559. doi: 10.1016/j.ahjo.2025.100559 (PMC12182336; doi:10.1016/j.ahjo.2025.100559)
Supplement: Supplementary file 2 — Supplementary material 2 [file mmc2.docx]

**Sensitivity Analysis**

For sensitivity analysis, we chose those studies in which the reported heterogeneity was greater than 75%.

The following risk factors were found to have high heterogeneity: Tumor size (86%), LDL (87%), Age (81%), Tumor Attachment Size (80%), BMI (94%), Hemoglobin (76%), Left Atrial Diameter (96%), Platelet count (90%). Studies in each risk factor analysis showing a different trend were identified and a leave-one-out analysis was performed for these risk factors. As shown in the table below,

| Risk Factor | No. of studies | Initial I^2^, Ph | Study removed | Final I^2^, Ph | Final P value |
| --- | --- | --- | --- | --- | --- |
| Hb | 3 | 76%, 0.02 | Kalçık 2019 | 29%, 0.23 | 0.04 |
| Tumor size | 10 | 86%, <0.00001 | Kalçık 2019 | 78%, <0.0001 | Unchanged |
| BMI | 3 | 94%, <0.00001 | Yin 2016 | 0%, 0.68 | Unchanged |
| LAD | 5 | 96%, <0.00001 | Yin 2016 | 37%, 0.19 | Unchanged |
| Age | 16 | 81%, <0.00001 | Yin 2016 | 72%, <0.00001 | Unchanged |
| LDL | 3 | 87%, 0.0004 | Gao 2014 | 65%, 0.09 | Unchanged |
| Platelet count | 6 | 90%, <0.00001 | Canga 2017 | 0%, 0.61 | <0.0001 |
| Attachment size* | 2 | 80%, 0.03 | - | - | - |

Table 4: Heterogeneity analysis for risk factors in embolism studies. Hb = Hemoglobin; BMI = Body Mass Index; LAD = Left Atrial Diameter; LDL = Low-Density Lipoprotein; I² = Heterogeneity statistic; Ph = P-value for heterogeneity.

Hb level and platelet count become significantly different in two groups after performing a leave one out analysis. (will add to discussion)

**Egger’s test**

Gender

Regression Test for Funnel Plot Asymmetry

Model: weighted regression with multiplicative dispersion

Predictor: standard error

Test for Funnel Plot Asymmetry: t = 1.5870, df = 13, p = 0.1365

Limit Estimate (as sei -> 0): b = -1.0386 (CI: -1.5537, -0.5235)

Irregular surface

Regression Test for Funnel Plot Asymmetry

Model: weighted regression with multiplicative dispersion

Predictor: standard error

Test for Funnel Plot Asymmetry: t = 2.2854, df = 12, p = 0.0413

Limit Estimate (as sei -> 0): b = 0.0495 (CI: -0.2836, 0.3825)

Age

Regression Test for Funnel Plot Asymmetry

Model: mixed-effects meta-regression model

Predictor: standard error

Test for Funnel Plot Asymmetry: z = -2.0797, p = 0.0375

Limit Estimate (as sei -> 0): b = 2.1470 (CI: -2.5827, 6.8766)

Tumor size

Regression Test for Funnel Plot Asymmetry

Model: mixed-effects meta-regression model

Predictor: standard error

Test for Funnel Plot Asymmetry: z = 0.3237, p = 0.7462

Limit Estimate (as sei -> 0): b = -0.3010 (CI: -1.8684, 1.2664)
